# Development of shielding evaluation and management program for O-ring type linear accelerators

**DOI:** 10.1038/s41598-024-60362-6

**Published:** 2024-05-10

**Authors:** Dong Hyeok Choi, So Hyun Ahn, Dong Wook Kim, Sang Hyoun Choi, Woo Sang Ahn, Jihun Kim, Jin Sung Kim

**Affiliations:** 1 Department of Medicine, Yonsei University College of Medicine, Seoul, Korea; 2 Department of Radiation Oncology, Yonsei Cancer Center, Heavy Ion Therapy Research Institute, Yonsei University College of Medicine, Seoul, South Korea; 3 Ewha Medical Research Institute, School of Medicine, Ewha Womans University, Seoul, South Korea; 4 Department of Radiation Oncology, Institute of Radiological and Medical Sciences, Seoul, Republic of Korea; 5 Department of Radiation Oncology, Gangneung Asan Hospital, University of Ulsan College of Medicine, Gangneung, Republic of Korea; 6 Department of Radiation Oncology, Gangnam Severance Hospital, Yonsei University College of Medicine, Seoul, Republic of Korea

**Keywords:** DICOM-RT, Shielding evaluation, Halcyon, Unity, Tomotherapy, Health care, Medical research, Oncology, Risk factors

## Abstract

The shielding parameters can vary depending on the geometrical structure of the linear accelerators (LINAC), treatment techniques, and beam energies. Recently, the introduction of O-ring type linear accelerators is increasing. The objective of this study is to evaluate the shielding parameters of new type of linac using a dedicated program developed by us named ORSE (O-ring type Radiation therapy equipment Shielding Evaluation). The shielding evaluation was conducted for a total of four treatment rooms including Elekta Unity, Varian Halcyon, and Accuray Tomotherapy. The developed program possesses the capability to calculate transmitted dose, maximum treatable patient capacity, and shielding wall thickness based on patient data. The doses were measured for five days using glass dosimeters to compare with the results of program. The IMRT factors and use factors obtained from patient data showed differences of up to 65.0% and 33.8%, respectively, compared to safety management report. The shielding evaluation conducted in each treatment room showed that the transmitted dose at every location was below 1% of the dose limit. The results of program and measurements showed a maximum difference of 0.003 mSv/week in transmitted dose. The ORSE program allows for the shielding evaluation results to the clinical environment of each institution based on patient data.

## Introduction

The linac and its software have been continuously advancing to meet the needs of clinical environments aiming to reduce treatment time and delivering an accurate dose to patients. These advancements include the increasing adoption of O-ring type linac. The O-ring type linac differs from c-arm type linac in terms of their structural features^1–3^. By housing the gantry head inside the bore, the O-ring type linac mitigates the risk of patient collision during rotation, resulting in increased gantry rotation speed^4–7^. Furthermore, the adoption of FFF beams and high leaf speeds in O-ring type linacs offers improved delivery efficiency and shorter treatment durations^1,3,8,9^. The advantages of maintaining treatment quality while acquiring images and its suitability for adaptive therapy have contributed to the increasing utilization of such linac^10–20^.

The distinctive feature of an O-ring type linac is its beam stopper structure, which attenuates the primary beam and saves construction costs and space^2^. The beam stopper typically adopts a rectangular configuration, possessing an area sufficient to cover the entire irradiation field of the primary beam. Its transmission rate attenuates the primary beam approximately by 0.1%, 0.6%, and 0.01% for Halcyon, Tomotherapy, and UNITY, respectively^21–23^. An assessment of shielding design for facility using linac requires an approach that takes into account such structural differences^24–28^. While the beam stopper notably reduces the intensity of the primary beam, it doesn’t entirely eliminate it, thus contributing to the leakage dose. This necessitates the application of the two-source formula to the primary barrier, which contrasts with the approach for standard linear accelerators^29^. As a result, for O-ring type linear accelerators, significant consideration must be given to both primary and secondary shielding.

In the clinical domain, domestic regulatory agencies have recently issued warnings against exceeding prescribed workload standards and advocated for the reevaluation of workloads^30^. The majority of radiotherapy facilities nationwide have adhered to the standard recommendations for shielding design provided by relevant international organizations, such as the NCRP-151 guidelines. Moreover, these facilities have adopted a more conservative standard for permissible weekly radiation doses compared to the guidelines proposed by the National Nuclear Safety and Security Commission (1 mSv/week for public access areas and 0.1 mSv/week for public areas)^31^. The establishment of a standardized system for each radiation-utilizing facility is imperative, and the methodology employed must be disseminated as fundamental data^32^. While it may be pragmatic for institutions with existing designs and shielding to revise their shielding design and structure in alignment with updated guidelines, this practice holds the potential to enhance shielding in radiation oncology departments that are either newly inaugurated or undergoing expansion with the incorporation of additional devices.

Using DICOM RT Plan files for shielding evaluation has several advantages over other methods. DICOM RT Plan files contain both treatment planning and dose distribution information, which ensures high accuracy in shielding evaluation. Furthermore, users can intuitively understand the information required to perform the evaluation, making it easier to understand the evaluation results. When performing shielding evaluations using this method, reproducibility can be improved because the evaluation can always be performed consistently over long-term monitoring.

Previous studies have focused on the use of DICOM RT Plan files for shielding evaluation in c-arm type linac^28,33^. However, there have been no instances of developed shielding evaluation programs for O-ring shaped linac yet. In this study, we developed and validated a program that automatically performs shielding evaluation using DICOM RT Plan files of newly introduced O-ring type linac. This approach represents a unique contribution to the field and has significant potential for clinical application. This program can help ensure the safety of patients and staff in radiation therapy facilities.

## Materials and methods

**Figure 1:**
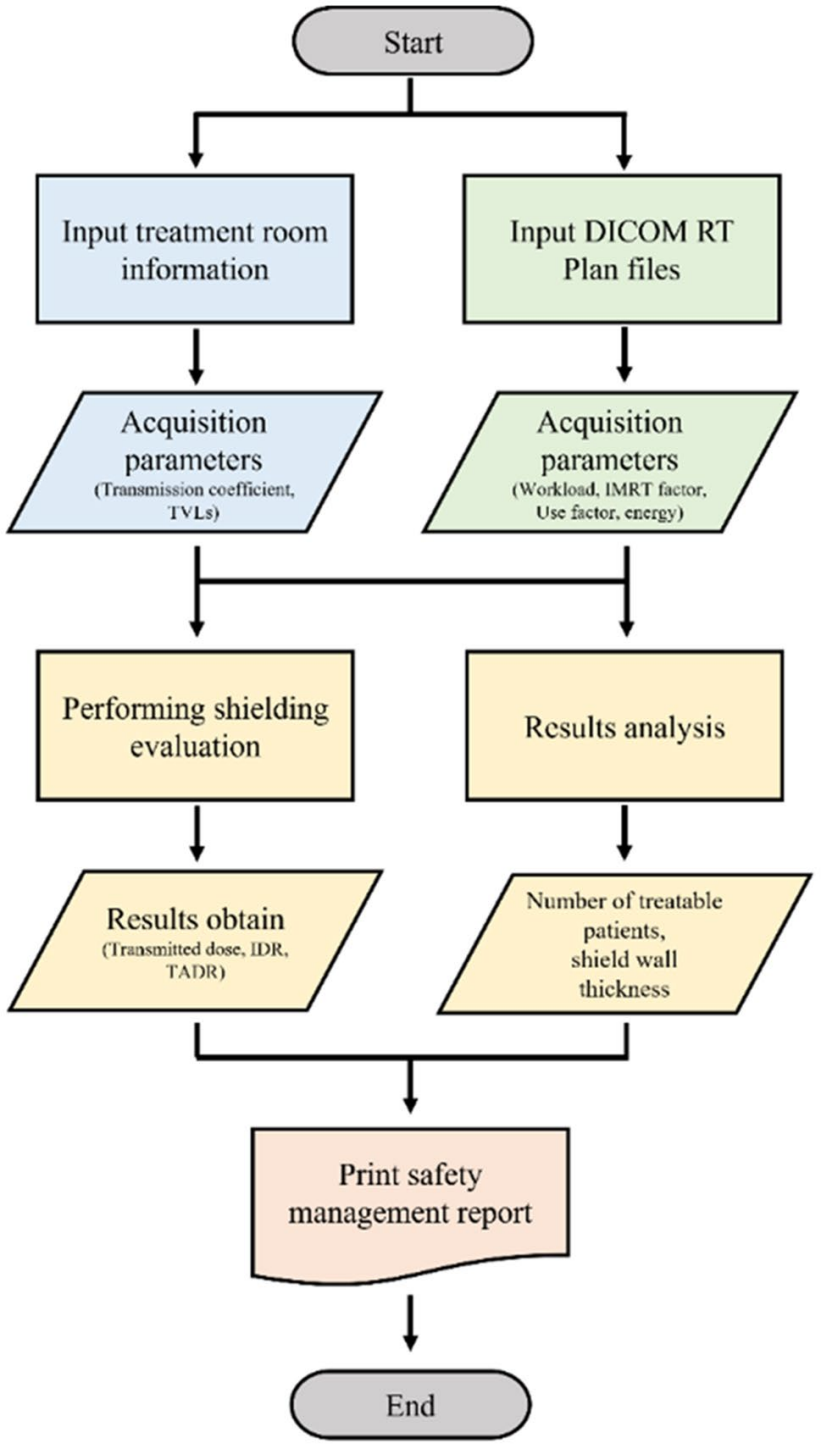
Flowchart of ORSE program. The input data for the program consists of treatment room information and patient data, which are used to perform shielding evaluation and ultimately generate a treatment room safety report.

The ORSE program was developed using MATLAB to evaluate the shielding parameters from patient’s DICOM RT plan file. The flowchart of the program is shown in Fig. 1. In order to run the ORSE program, the required input files are (1) file containing shielding information of the treatment room as comma-separated values (CSV) format, and (2) DICOM RT plan files of patients who received treatment during the desired analysis period. These two kind of files are to be stored in a same folder, and when loaded into the ORSE program, the shielding factors are calculated and saved in a report format.

### Calculation of shielding parameters

The ORSE program evaluates shielding by leveraging the NCRP-151 formula and calculating the transmitted doses of the primary and secondary beams at six designated points outside the treatment room. Despite the beam stopper’s significant attenuation of the primary beam’s intensity, it doesn’t completely nullify it, thereby contributing to the leakage dose. This characteristic necessitates the ORSE program’s application of the two-source formula to the primary barrier. This particular attribute necessitates the application of the two-source formula to the primary barrier within the ORSE program. Consequently, when addressing O-ring type linear accelerators, the program mandates an intensive focus on both primary and secondary shielding. The program also calculates the workload, use factor, and IMRT factor required using the patient’s DICOM RT plan file. The corresponding DICOM RT plan file to account for the QA beam usage is included in the shielding evaluation process.

#### Use factor

The ORSE program calculates the use factor by classifying the MU information for each control point of each field of the DICOM RT Plan file and determining the ratio of the MU value of a specific angle to the total MU. The gantry angles of 0°, 90°, 180° and 270° correspond to both wall, floor and ceiling directions, and the use factors are corrected at 90° intervals for shielding calculation. For example, for an angle of 180°, the weight of the MU sum of 135° to 225° is used.

To compare the use factor obtained by the method proposed by Kaur^22^ and the use factor obtained in this study, the use factors were obtained for each of the two Tomotherapy. This effort is to compare the method used to induce the use factor with previous research methods and will be described in the discussion section.

#### IMRT factor

The IMRT factor is defined as the beam usage required for 3D-CRT treatment ${MU}_{3DCRT}$ compared to the beam usage required for IMRT treatment ${MU}_{IMRT}$ to deliver the same prescribed dose.

1 $$ IMRTfactor \left( {IF} \right) = \frac{{MU_{IMRT} }}{{MU_{3DCRT} }} $$

Using the formula presented in the NCRP 151 report, calculate the $MU_{IMRT}$ of Eq. (1) using the prescription dose and MU obtained from the patient DICOM RT Plan file.

2 $$ MU_{IMRT} = \mathop \sum \limits_{i} \frac{{MU_{i} }}{{\left( {Dose_{pre} } \right)_{i} }} $$

where $i$ means the number of different DICOM RT plan files. Defined as the MU ratio of IMRT and 3D-CRT required to deliver the same absorbed dose in the beam area to the phantom installed at source-to-axis distance 100 cm. To take this into account, the program calculates the MU value using the Tissue maximum ratio (TMR) data from each hospital.

3 $$ MU_{IMRT} = \mathop \sum \limits_{i} \frac{{MU_{i} }}{{\left( {Dose_{pre} \times TMR} \right)_{i} }} $$

The IMRT factor of the Tomotherapy is calculated using the dose rate and the treatment time as shown in Eq. (4). Therefore, to calculate the IMRT factor for Tomotherapy, the unique dose rate information for each linac should be provided in a CSV file.

4 $$ MU_{IMRT \cdot Tomo} = \mathop \sum \limits_{i} \frac{{Time_{i} }}{{\left( {Dose_{pre} \times TMR} \right)_{i} }} \times Dose\;Rate \left( {\frac{cGy}{{min}}} \right) $$

The IMRT factor of the Tomotherapy is calculated by replacing the $MU_{IMRT}$ of Eq. (1) with $MU_{IMRT \cdot Tomo}$ of Eq. (4).

### Treatment room management function

#### Maximum number of treatable patients

Calculate the maximum number of patients that can be treated using the workload planned in the safety management report of the treatment room and the workload acquired through patient data. The maximum number of treatable patients is calculated by Eq. (5).

5 $$ Maximum\;treatable\;patients = Worklaod_{{\left( {planned} \right)}} / Workload_{{\left( {pt} \right)}} $$

where $Worklaod_{{\left( {planned} \right)}}$ mean to the planned weekly operational load documented in the safety management report and $Worklaod_{{\left( {pt} \right)}}$ mean the value obtained by dividing the total workload of treated patients by the number of patients.

An example of calculating the maximum treatable patient is as follows: When designing shielding facilities, the planned weekly workload ($Worklaod_{{\left( {planned} \right)}}$ from the safety management report is determined to be $300{\text{ cGy}}/{\text{patient }} \times { }50{\text{ patients}}/{\text{day }} \times { }5{\text{ days}}/{\text{week }} \times { }5{ }\left( {\text{IMRT factor}} \right),{ }$ totaling 375,000 cGy per week. Assuming an average prescription dose of 250 cGy per patient $(Workload_{{\left( {pt} \right)}} )$ with an IMRT factor of 3, the weekly dose per patient is 3750 cGy. Dividing the planned weekly workload ($Worklaod_{{\left( {planned} \right)}}$) by the per-patient workload ($Workload_{{\left( {pt} \right)}}$), the calculation yields a capacity of 100 patients.

#### Optimize shield wall thickness

The ORSE program can propose the optimizing thickness of the barrier wall based on the beam energy and the workload of the treated patient. This allows for easy comparison with the thickness of the shielding wall based on the concrete thickness in the safety management report created during the design of the treatment room.

#### Safety management report

The ORSE program can generate a safety management report of the treatment room in CSV file format. This report includes various items such as the result of analyzing the DICOM RT plan file, the shielding evaluation result, the patient number management result, and the shielding wall thickness optimization result.

### The operation and verification of the ORSE program

The ORSE program was conducted on a total of four O-ring type linac for three institutions. The specifications of Unity, Halcyon, and Tomotherapy are shown in Table 1.

**Table 1:** The information of imaging method, manufacturer, energy, treatment technology, maximum radiation dose, and beam stopper for each treatment room.

Equipment	Manufacturer	Energy	Source-to-isocenter distance	Treatment technique	Maximum output dose rate	Beam stopper
Unity	Elekta	7 MV FFF	143.5 cm	IMRT, SRS, SBRT, 3D-CRT	700 cGy/min	Pb + steel (15.2 + 1.8 cm)
Halcyon	Varian	6 MV FFF	100 cm	IMRT, VMAT, 3D-CRT	800 cGy/min	Pb + steel (17.2 + 2.0 cm)
Tomotherapy	Accuracy	6 MV FFF	85 cm	Helical IMRT, 3D-CRT	850 cGy/min	Pb (12.7 cm)

We compared the measured transmitted dose with the shielding evaluation results obtained using ORSE to verify the results of the penetration dose obtained using the program. The detector (Chiyoda Technol., Japan) containing a glass dosimeter for photon detection was used, which was enclosed in a plastic package for measuring the cumulative dose in the treatment room. The glass dosimeter was calibrated using Cs-137 γ rays as a standard irradiation field for X·γ rays. The glass dosimeter has an uncertainty of up to 1.25% in the range of 10–100 keV. The measurement was carried out for the same period, 5 days, during which the patient DICOM RT plan file was collected. Two detectors were installed at each of the four points in each treatment room in all the pilot operation institutions, with one location in the primary beam direction and three locations in the secondary beam. The dose was found to be below the detection limit when the detector was placed on the outer wall of the treatment room. Therefore, We the detector was placed on the inner wall of the treatment room. The transmission dose was calculated by the occupancy factor and the transmission coefficient of the shielding barrier to the cumulative dose of the detectors.

#### Information of treatment room

**Figure 2:**
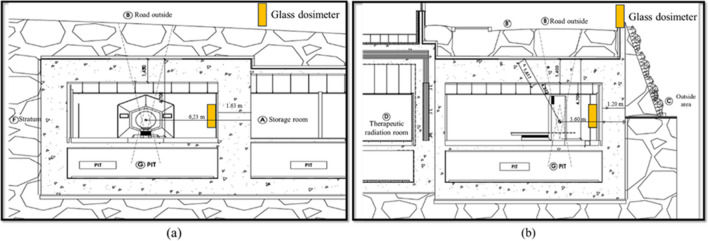
Drawing of the Halcyon treatment room from two directions. (**a**) A glass dosimeter installed on the primary beam wall (**b**) A glass dosimeter installed on the secondary beam wall.

The safety management report for each treatment room were collected to gather the information on each treatment room in the three institutions. The information includes the distance between the isocenter and the evaluation point, occupancy factor, material and thickness of the shielding wall, and factors necessary for performing scattering line and shielding calculations of the door. Additionally, beam stopper information was obtained to calculate the transmitted dose for the primary beam. The user can insert the information about the usage of the QA beam to consider the QA beam. And the existing DICOM RT plan file can be replaced with a new RT plan file for including the adaptive treatments. All the information collected from the safety management report was entered into a CSV file format for execution of the program. Figure 2 shows a geometry of the Halcyon treatment room and shows an example of where to install the glass dosimeter.

#### Patients data

We manually collected DICOM RT plan files of patients who underwent treatment for a duration of five working days in August 2021. A total of 111 patients were gathered from four treatment rooms. Personal identifiers, including name, age, and gender, were anonymized to safeguard patient privacy.

Without institutional review board (IRB) approval, including exemption approval for consent from research participants, it is not permissible to utilize anonymized personal information from research subjects without their individual consent, despite compliance with privacy laws. Therefore, in this study, IRB approval was obtained for the collection and utilization of patient data for research purposes regarding ethics approval for data collection and utilization.

Each institution obtained approval of the IRB before collecting patient data Gangnam Severance Hospital (4-2021-0857), Yonsei Cancer Center (4-2022-1488), Gangneung Asan Hospital (2021-04-034). Due to the retrospective nature of this study, which involved the collection of data from previously treated patients, the acquisition of patient consent was deemed unnecessary in compliance with established guidelines and regulations. All patient data were anonymized, and all methods were performed in accordance with the relevant guidelines and regulations.

## Results

### Execution of ORSE

**Figure 3:**
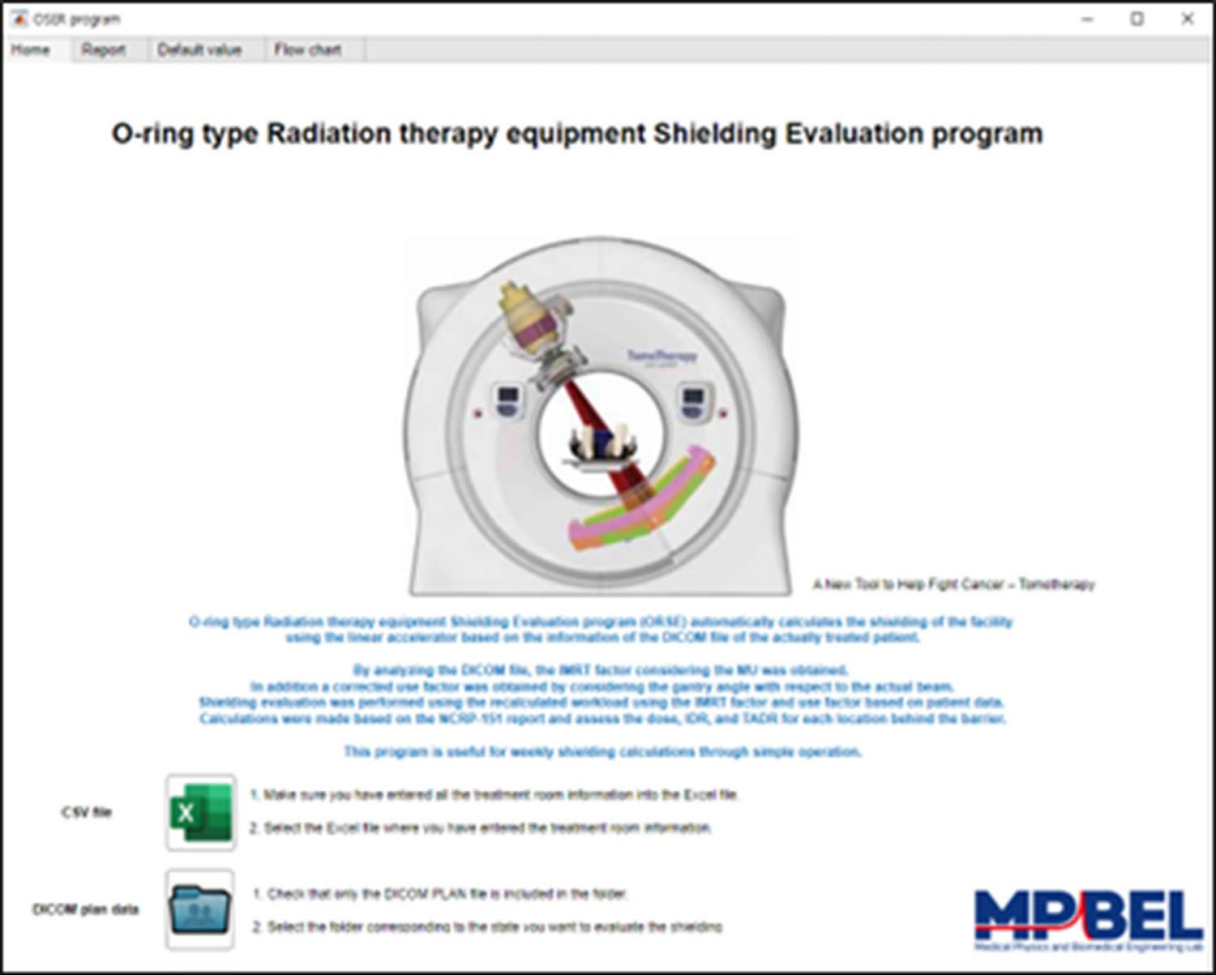
Home screen of ORSE: Buttons for loading CSV file (Green) and DICOM RT plan file (Blue) are shown at the bottom.

The ORSE program consists of four tabs such as (1) Home, (2) Report, (3) Default value, and (4) Flow chart. The Home screen includes buttons for inputting treatment room information and patient data. After entering and saving the treatment room information, the program automatically outputs the shielding evaluation and treatment room management report. The entered data can be confirmed in the Default tab, and the program flow can be checked in the Flow chart tab. The Home screen layout is shown in Fig. 3. The program execution time varies depending on the computer specifications, but it takes an average of approximately three minutes. For a PC with an AMD Ryzen 9 5900X, it takes less than 2 min.

### Results of ORSE

The ORSE program provides 5 results which can be viewed in the Report tab: (1) DICOM data analysis, (2) shielding evaluation of the treatment room, (3) graph of transmitted dose compared to dose limit, (4) number of treatable patients, and (5) optimization of shielding wall thickness. The DICOM data analysis tab displays workload, IMRT factor, use factor, the number of patients according to treatment techniques and energies, obtained from DICOM RT plan files. The program distinguishes treatment techniques based on the number of fields and gantry rotation information for each patient data, and outputs the assigned parameters. The numerical values are displayed on the right of each result for comparison with safety management report values.

The Treatment Room Analysis tab shows the transmitted dose at points around the treatment room, the maximum number of patients that can be treated per day and per week, and the shielding wall thickness based on the workload.

#### DICOM file analysis

##### IMRT factor

**Figure 4:**
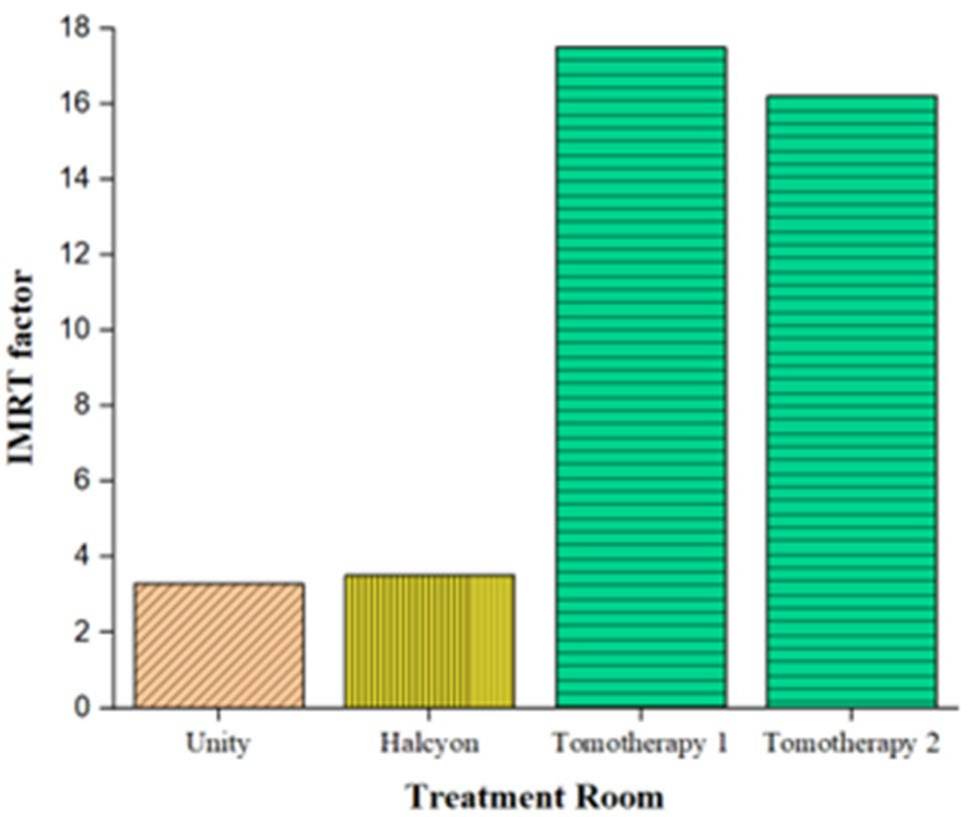
IMRT factor for each treatment room.

The values of IMRT factor used in safety management report vary across institutions, with Unity at 5, Halcyon at 10, and Tomotherapy at 15. Figure 4 presents the results of the IMRT factor analysis by treatment room, obtained from the DICOM RT plan file. Each bar graph represents the IMRT factor obtained from each institution/linac, as well as the reference value of IMRT factor in the safety management report. Treatment rooms using Unity and Halcyon had IMRT factors that were 34% and 65% lower than the values in the safety management report, respectively. On the other hand, the treatment room using Tomotherapy had IMRT factors that were 17% and 8% higher than the values in the safety management report, respectively.

##### Use factor

The results of the analysis of the use factor for each treatment room based on the DICOM RT plan files are presented in Table 2. The calculated use factors by ORSE showed an average difference of 15.8 ± 8.7% compared to safety management report, with a maximum difference of 33.5%.

**Table 2:** The use factor for each treatment room calculated using ORSE.

Linac	Gantry angle	ORSE results	Safety management report	%difference
Unity	0°	0.278	0.310	− 10.32
	90°	0.245	0.213	15.02
	180°	0.236	0.263	− 10.27
	270°	0.241	0.213	13.15
Halcyon	0°	0.354	0.310	14.19
	90°	0.258	0.213	21.13
	180°	0.175	0.263	− 33.46
	270°	0.213	0.213	0.00
Tomo 1	0°	0.235	0.310	− 24.19
	90°	0.257	0.213	20.66
	180°	0.254	0.263	− 3.42
	270°	0.254	0.213	19.25
Tomo 2	0°	0.233	0.310	− 24.84
	90°	0.256	0.213	20.19
	180°	0.255	0.263	− 3.04
	270°	0.256	0.213	20.19

##### Workload

The workload for primary and secondary radiation were obtained using the ORSE program. The workload for secondary beams was calculated by reflecting IMRT factors obtained through the ORSE program. The results of the workload for each treatment room are available in Table 4.

##### Ratio of treatment techniques and beam energies

The Unity uses only IMRT treatment technique with 7 MV FFF beam. The Halcyon uses a 62% IMRT treatment technique and a 38% VMAT treatment technique with 6 MV FFF beam. Tomotherapy uses only VMAT treatment technique with 6 MV FFF beam.

#### Shielding evaluation

Table 3 shows the types of beams, dose limits, measured doses, doses calculated by ORSE, and expected doses in safety management report for each institution’s treatment room. For measurement purposes, glass dosimetry was installed on the four walls and in the direction of the entrance door, excluding the ceiling and floor where installation was deemed impractical. The maximum difference between the predicted transmitted dose outside the shielding wall from the calculated dose by ORSE and the measured dose was about 0.003 mSv/week.

**Table 3:** Radiation therapy room management.

linacs	Points	Type of beams (mSv/week)	Doses (mSv/week)	Dose limits		
				Measured by detectors	Calculated by ORSE	Safety management report
Unity	1	Primary	0.4	0.00057	0.00104	0.00056
	2	Primary	0.4	0.00027	0.00063	0.00056
	3	Secondary	0.02	0.00089	0.00032	0.00524
	4	Secondary	0.02	0.00094	0.00151	0.00564
Halcyon	1	Primary	0.4	< 0.00001	0.00026	0.00015
	2	Secondary	0.02	0.00024	0.00059	0.00360
	3	Secondary	0.02	0.00012	0.00006	0.00023
	4	Secondary	0.02	0.00008	0.00011	0.00011
Tomo 1	1	Primary	0.02	< 0.00001	0.00913	< 0.00001*
	2	Secondary	0.02	< 0.00001	0.00322	< 0.00001*
	3	Secondary	0.4	0.00052	0.00295	0.00014
	4	Secondary	0.4	0.00113	0.00112	0.01889
Tomo 2	1	Primary	0.02	0.00003	0.00101	0.00005
	2	Secondary	0.4	0.00941	0.00610	0.02033
	3	Secondary	0.02	0.00157	0.00058	0.00191
	4	Secondary	0.4	0.00031	0.00109	0.02010

*Values smaller than 0.00001 in the radiation safety report are expressed as < 0.00001 for consistency in decimal point notation.

#### Radiation therapy room management

When executing the ORSE program, results such as shielding evaluation factors obtained through DICOM files, transmitted dose results at each point, the maximum number of treatable patients, and shielding wall thickness based on workload are automatically saved and output as a CSV file.

##### Maximum number of treatable patients

All four treatment rooms could accommodate more patients than currently treated. In particular, the Halcyon was able to treat more than four times the planned number of patients calculated using the current IMRT factor of 10 (Table 4).

**Table 4:** The maximum number of treatable patients per treatment room calculated through the ORSE program.

Equipment	Item	ORSE results	Safety management report
Unity	Number of patients	18 (C)	80
	Workload (Gy/week)	833 (B)	6000 (A)
	Calculate: (A)/(B/C) = 129		
	Maximum number of treatable patients	129	
Halcyon	Number of patients	49	100
	Workload (Gy/week)	1773	15,000
	Maximum number of treatable patients	405	
Tomo 1	Number of patients	22	40
	Workload (Gy/week)	4242	9000
	Maximum number of treatable patients	46	
Tomo 2	Number of patients	22	40
	Workload (Gy/week)	3912	9000
	Maximum number of treatable patients	50	

##### Optimization of shielding wall thickness

The optimizing thickness of the shielding wall was calculated based on the workload obtained through the ORSE program using concrete as a reference material. The program results were compared with the material and thickness of the shielding wall obtained through a safety management report (Table 5). In addition, the thickness of each material in the safety management report was converted to concrete thickness for easy comparison. The shielding wall in the safety management report was designed to be conservative with a concrete thickness that is more conservative than the program results, aiming for a transmitted radiation dose of less than 10% compared to the dose limit based on the radiation use plan.

**Table 5:** The results of the optimizing shielding wall thickness.

Equipment	Point	Beam type	Shield wall thickness (cm)				
			ORSE results	Safety management report			
			Concrete	*Concrete	Concrete	Steel	Lead
Unity	1	Primary	76.5	165.8	79.0	–	15.0
	2	Primary	75.1	165.8	79.0	–	15.0
	3	Secondary	7.0	148.9	120.0	–	5.0
Halcyon	1	Primary	38.8	262.6	163.0	2.0	17.2
	2	Secondary	26.4	120.0	163.0	–	–
	3	Secondary	14.9	124.4	140.0	–	–
Tomo 1	1	Primary	24.8	315.3	240.0	–	13.0
	2	Secondary	13.6	240	240.0	–	–
	3	Secondary	66.5	100	100.0	–	–
Tomo 2	1	Primary	85.2	212.6	120.0	–	16.0
	2	Secondary	64.3	120.7	120.7	–	–
	3	Secondary	13.9	149	149	–	–

*The thickness of the multi-layer shielding for various materials in the safety report is converted into concrete thickness.

## Discussion

There have been studies that performed shielding evaluation using DICOM RT plan files in previous research^28,33^. However, those studies evaluated only c-arm type linac, and there was no program design or evaluation for O-ring type linac, making this study innovative in this regard. In addition, unlike the method proposed in previous studies, it is possible to take into account QA beams and perform shielding evaluation that reflects the structure of O-ring type linac.

In this study, the proposed ORSE program enabled shielding evaluation that takes into account the clinical environment of treatment rooms using O-ring type linac. This is possible by analyzing the information used to treat actual patients in the DICOM RT Plan. There are studies that have calculated the use factor through mathematical calculation^22,29,33^. To perform these calculations, information such as the source to axis distance (SAD), the distance of the point of interest (POI) from the isocenter, and the angle information of the target corresponding to the maximum field size opened from the isocenter is required. Consequently, precise calculation of the use factor is attainable. However, in this study, a similar approach is employed as in previous research that computed the use factor using information from the DICOM RT Plan file^28,34^. The DICOM RT Plan file contains data on gantry angles and corresponding MU at those angles. Obtaining the use factor through this method not only applies to treatment techniques but also possesses the capability to reflect the dose delivered at specific angles depending on the treatment site.

The ORSE program can be used for various purposes. In cases where one wants to analyze patient data over a specific period, all data from patients treated during the evaluation period can be collected in one folder and used as a real-time monitoring program for shielding evaluation by collecting only patient data. Additionally, by collecting only patient data treated with a specific treatment technique, the extent to which it affects the safety management within the treatment room can be evaluated on a per-technique basis. When evaluating data collected from patients treated with the same technique, there is a risk of increased transmitted radiation at specific points. Based on the transmission dose obtained from each location through the ORSE program, it can be utilized as a function to divide patients receiving treatment in a treatment room with a high concentration of specific treatment sites into other treatment rooms. The ORSE program can also be used in designing initial radiation therapy rooms. By collecting patient data corresponding to the number of patients targeted for treatment per week in the treatment room and in accordance with the ratio of the treatment technique that one wishes to perform, one can determine an appropriate shielding wall thickness by obtaining the transmitted dose through the program.

By attaching two detectors to each of the four selected locations in each treatment room, we conducted measurements to obtain actual data, which were then compared with the calculated radiation transmission results for each location using the ORSE program. Excluding the results below the minimum detectable activity, the difference between the ORSE program results and the measured values was an average of 0.00078 ± 0.00095 mSv, with a maximum difference of 0.00331 mSv. This difference is expected to be caused by the detection error and direction dependence of the detector itself. Halcyon was designed conservatively by setting the IMRT factor to “10” when designing the shielding wall in our institution. However, analyzing data of patients treated with Halcyon through the ORSE program resulted in obtaining an IMRT value up to 65% lower than that in the safety management report. This indicates that the shielding wall was designed very conservatively, but it also implies that excessive shielding was designed. In such situations, the ORSE program can optimize the thickness of the shielding wall based on the workload by using actual patient data.

Kaur et al.^22^ proposed a simple mathematical formula for determining the use factor of tomotherapy. Previous study derived mathematical expressions for the angle of divergence of the beam from the source corresponding to the field length, the angle of rotation of the source with respect to the isocenter, and the distance of the barrier from the isocenter to calculate use factor. The equations are as follow Eq. (6) and (7).

6 $$ \alpha = 2tan^{ - 1} \left( {\left[ {1 + \frac{R}{d}} \right]tan\theta /2} \right) $$

7 $$ Use\, factor = \frac{\alpha }{{360^{^\circ } }} $$

where $\alpha$ is the angle subtended at the isocenter by the central axes of beams corresponding to the target. And $\theta$ is the angle corresponding to the X-ray target/source in the gantry rotation plane at the isocenter of the field size, R is the distance of the X-ray target from the isocenter, and d is the distance from the isocenter to the point of interest on the primary barrier.

In this study calculated the use factor in a way similar to that of a linear accelerator to compare the use factor derived from the DICOM RT Plan file with the one used in the safety management report. In the safety management report, the use factor is used at intervals of 90 degrees, and in this study, the use factor was calculated based on the MU value within the range of 90 degrees based on each angle. In this study, the use factor of Tomotherapy ranged 0.233 to 0.257. Conversely, the range of use factors obtained for the four angles utilizing the method proposed by Kaur et al. was 0.108–0.170. In the case of our method, there is a point that the patient’s DICOM RT Plan file is essential. If the DICOM RT Plan file is difficult to obtain, Kaur et al.’s method can be useful, and a simple formula provides excellent accessibility.

This study has limitations in that it does not incorporate treatment techniques such as SRS, SBRT, and TBI. It also does not account for specific treatment fractions that cannot be received due to a patient’s special circumstances or linac malfunction. To overcome these limitations, we aim to develop a program that can analyze various treatment techniques. Additionally, we plan to supplement the program by reflecting cases where a patient cannot receive treatment on a specific date through integration with an Electronic Medical Record (EMR) system and outputting the shielding evaluation results^22^.

The results of this study demonstrate the need to re-evaluate shielding evaluation factors for O-ring type linac and are significant in that they can be used to analyze trends in shielding evaluation results for each treatment room considering the clinical environment (Supplementary information).

## Conclusion

The shielding management program based on patient DICOM RT plans, ORSE, is much more useful and efficient than the conventional manual calculation and management method for shielding evaluation. In addition, it can easily perform shielding evaluation using patient data for a desired period of time, and can be efficiently applied to radiation safety management in treatment rooms.

### Supplementary Information

Supplementary Information.

## Data Availability

The datasets generated during and/or analyzed during the current study are available from the corresponding author on reasonable request.
