# Author Correction: Development of shielding evaluation and management program for O-ring type linear accelerators

**DOI:** 10.1038/s41598-024-62453-w

**Published:** 2024-05-21

**Authors:** Dong Hyeok Choi, So Hyun Ahn, Dong Wook Kim, Sang Hyoun Choi, Woo Sang Ahn, Jihun Kim, Jin Sung Kim

**Affiliations:** 1 Department of Medicine, Yonsei University College of Medicine, Seoul, Korea; 2 Department of Radiation Oncology, Yonsei Cancer Center, Heavy Ion Therapy Research Institute, Yonsei University College of Medicine, Seoul, South Korea; 3 Ewha Medical Research Institute, School of Medicine, Ewha Womans University, Seoul, South Korea; 4 Department of Radiation Oncology, Institute of Radiological and Medical Sciences, Seoul, Republic of Korea; 5 Department of Radiation Oncology, Gangneung Asan Hospital, University of Ulsan College of Medicine, Gangneung, Republic of Korea; 6 Department of Radiation Oncology, Gangnam Severance Hospital, Yonsei University College of Medicine, Seoul, Republic of Korea

Correction to: *Scientific Reports* 10.1038/s41598-024-60362-6, published online 10 May 2024

The Acknowledgments section in the original version of this Article was incomplete.

“This work was supported by a faculty research grant of Yonsei University College of Medicine (6-2021-0234), and by the Nuclear Safety Research Program (RS-2022-KN071210) through the Korea Foundation of Nuclear Safety (KOFONS) using the financial resource granted by the Nuclear Safety and Security Commission (NSSC) of the Republic of Korea, and by Basic Science Research Program through the National Research Foundation of Korea (NRF) funded by the Ministry of Education (RS-2023-00240003).”

now reads:

“This work was supported by a faculty research grant of Yonsei University College of Medicine (6-2021-0234), by Korea Institute for Advancement of Technology (KIAT) grant funded by the Korea Government (P0026103) and by the Nuclear Safety Research Program (RS-2022-KN071210) through the Korea Foundation of Nuclear Safety (KOFONS) using the financial resource granted by the Nuclear Safety and Security Commission (NSSC) of the Republic of Korea, by Basic Science Research Program through the National Research Foundation of Korea (NRF) funded by the Ministry of Education (RS-2023-00240003).”

The original Article has been corrected.

